# West Nile Virus Restriction in Mosquito and Human Cells: A Virus under Confinement

**DOI:** 10.3390/vaccines8020256

**Published:** 2020-05-29

**Authors:** Marie-France Martin, Sébastien Nisole

**Affiliations:** Viral Trafficking, Restriction and Innate Signaling Team, Institut de Recherche en Infectiologie de Montpellier (IRIM), CNRS, 34090 Montpellier, France; marie-france.martin@irim.cnrs.fr

**Keywords:** West Nile virus, restriction factors, interferon, innate immunity, mosquito, viral countermeasures, viral evasion

## Abstract

West Nile virus (WNV) is an emerging neurotropic flavivirus that naturally circulates between mosquitoes and birds. However, WNV has a broad host range and can be transmitted from mosquitoes to several mammalian species, including humans, through infected saliva during a blood meal. Although WNV infections are mostly asymptomatic, 20% to 30% of cases are symptomatic and can occasionally lead to severe symptoms, including fatal meningitis or encephalitis. Over the past decades, WNV-carrying mosquitoes have become increasingly widespread across new regions, including North America and Europe, which constitutes a public health concern. Nevertheless, mosquito and human innate immune defenses can detect WNV infection and induce the expression of antiviral effectors, so-called viral restriction factors, to control viral propagation. Conversely, WNV has developed countermeasures to escape these host defenses, thus establishing a constant arms race between the virus and its hosts. Our review intends to cover most of the current knowledge on viral restriction factors as well as WNV evasion strategies in mosquito and human cells in order to bring an updated overview on WNV–host interactions.

## 1. Introduction

### 1.1. West Nile Virus Incidence 

West Nile virus (WNV) belongs to the Flaviviridae family, from the *Flavivirus* genus, which also comprises Zika virus (ZIKV), dengue virus (DENV), tick-borne encephalitis virus (TBEV), and yellow fever virus (YFV). All these viruses are transmitted by mosquitoes and are therefore known as arboviruses (for arthropod-borne viruses). WNV was first isolated in the West Nile district of Uganda in 1937 and has since spread across the world [1]. This enveloped virus has a 11-kb positive single-stranded RNA genome (ssRNA) that encodes three structural proteins (C, E, prM) and seven non-structural proteins (NS1, NS2A, NS2B, NS3, NS4A, NS4B, NS5). The viral genome is composed of only one single open reading frame (ORF), flanked by two untranslated regions (5′ and 3′ UTR). The flavivirus RNA genome is capped, but unlike cellular mRNA, it lacks a poly (A) tail. 

WNV, like the equally concerning Usutu virus [2,3], belongs to the Japanese encephalitis virus (JEV) serocomplex. It is maintained in nature in an enzootic cycle between mosquitoes of the *Culex* genus as vectors, and birds as the main reservoir and amplifier hosts. Although mammals can be infected during a mosquito blood meal, these are considered as dead-end hosts since viral replication ends rapidly (short and low viremia). Sometimes, mosquitoes that have fed on infected birds can then accidentally transmit the virus to humans. Although *Culex* mosquitoes are considered as the predominant vector for WNV, other mosquito genera, such as *Aedes* or *Ochlerotatus*, can also serve as bridge vectors [4].

WNV strains are divided into several distinct lineages based on the viral envelope protein [5,6]. Lineage 1 includes African (WN-CAR-1989), American (WN-New York-1999F), Australian (Kunjin), European (WN-Romania-1997; WN-France-2004), and Indian (WN-India-1980) strains, while lineage 2 is composed of strains present in a few countries, primarily in mainland Africa (WN-Senegal-1990) and in Madagascar (WN-Madagascar-1978) but also in Europe (WN-Hungary-2004; WN-Greece-2010) [7,8,9]. In addition, there are four other lineages: Lineages 3, 4, 5, and 6 that are only represented by few isolates [10,11,12,13]. 

Globalization and climate change are key factors of WNV transmission. Indeed, international traffic, as well as the increase in heavy rainfalls and warmer temperatures in temperate areas, such as North America and Europe, have a direct impact on the spread of mosquitoes to new ecological niches and host populations, and their ability to transmit WNV [14,15]. Thus, periodic WNV outbreaks are increasingly observed in Europe, highlighting the importance of surveillance strategies like those defined by the European Centre for Disease Prevention and Control (ECDC). 

For 20 years, some strains (Israel 2000 [16], New-York 1999 [17], Hungary 2004 [18]) have been responsible for important human outbreaks associated with high neurovirulence and leading to several human deaths. Indeed, 20%–30% of total cases of infection lead to a pathology known as West Nile fever (WNF), whose symptoms include fever, headache, or rash [19,20]. Among these symptomatic patients, less than 1% develop neurological complications referred to West Nile disease (WND) [8]. WND is lethal in 10% of the cases, and is often due to meningitis or encephalitis [19]. WNV neuroinvasion can be explained in part by the ability of WNV to cross the blood–brain barrier [21] and infect neurons, the spinal cord, and the brain stem [22,23]. More recently, in 2018, the greatest European WNV outbreak recorded to this day led to 2083 confirmed autochthonous human cases and 181 deaths [24].

These outbreaks highlight the importance of understanding WNV biology and the human immune response upon infection.

### 1.2. West Nile Virus Replication

**Figure 1:**
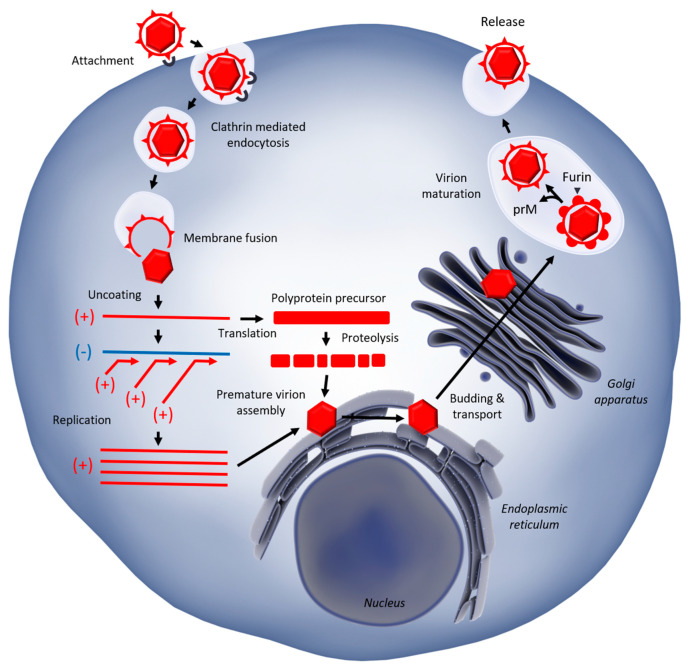
Replication cycle of West Nile Virus in host cells.

In human cells, the WNV replication cycle starts with viral envelope glycoproteins binding cellular receptors, such as the Dendritic Cell-Specific Intracellular adhesion molecule-3-Grabbing Non-integrin (DC-SIGN(R)), notably expressed by dendritic cells (DCs), [25] and probably integrins [26] (Figure 1). Viral entry is then mediated by clathrin-dependent endocytosis [27] and acidification, leading to membrane fusion and the release of the genetic material into the cytoplasm, where the viral RNA is translated as a polyprotein. The ensuing seven non-structural proteins (NS1A, NS1B, NS2A, NS2B, NS3, NS4A, NS4B, NS5) constitute the replication complex in which genomic RNA synthesis takes place. Then, the newly synthetized genomes are assembled together with structural proteins (capsid C, envelope E, and pre-mature membrane prM) at endoplasmic reticulum (ER) membranes to form pre-mature virions. After assembly, prM is cleaved by host cell furin in the trans-Golgi network to generate mature virions that are released by exocytosis. 

While this previous description describes the replicative cycle in the best conditions for the virus, different steps can be targeted by immune defenses to hamper viral replication.

Viral particles attach to cellular receptors at the plasma membrane via E protein binding. After virus attachment, entry occurs via clathrin-mediated endocytosis [27] followed by the acidification of endosomal vesicles. Endosomal acidification triggers viral fusion with the endosomal membrane [28] and the release of the (+) ssRNA genome into the cytoplasm after virion uncoating [29]. The viral genome is then translated into a single polyprotein, which is cleaved by viral proteases NS2B/NS3 and cellular proteases [30]. All NS proteins are part of the replication complex localized in replication organelles at the ER [31]. In these compartments, the viral RNA-dependent RNA polymerase NS5, along with others NS proteins, synthetizes a (-)ssRNA intermediate that then serves as a template for genomic (+)ssRNA synthesis. Structural proteins C, E, and prM assemble on the ER with genomic RNA to form immature virions. Immature viral particles traffic through the secretory pathway, resulting in host furin cleavage of prM into mature membrane protein M and glycosylation of viral envelope protein [32]. Finally, mature virions are released by exocytosis at the plasma membrane [33].

## 2. Mosquitoes: Immune Defenses against WNV Infection and Viral Countermeasures

### 2.1. Immune Response 

Our understanding of the mosquito innate immune system derives mainly from detailed studies in the *Drosophila melanogaster* model, whose immune system is well conserved with that of mosquitoes [34]. Mosquito innate immunity is mainly based on RNA interference (RNAi) pathways [35], which inhibit viral replication by RNA interference and secretion of the cytokine Vago [36,37]. These pathways involve small interfering RNAs (siRNA) [38], microRNAs (miRNA) [39], and P-element Induced Wimpy-interacting RNAs (PIWI-interacting RNAs, piRNA) [40]. In addition to RNAi, other mechanisms are also involved in innate defenses, including the toll, IMD (immune deficiency), and JAK/STAT pathways, which lead to the secretion of antimicrobial peptides.

Mosquitoes are the vectors, and therefore key players, of WNV transmission. Once they ingest an infected blood meal on a viremic host, viral replication begins in the mosquito midgut epithelial cells and viral particles disseminate into hemolymph circulation to reach muscles and the neural system. Salivary glands, which constitute the end-point tissue, carry high viral loads [41], which favors viral transmission during the next blood ingestion on a susceptible host [42]. 

The extrinsic incubation period, which is defined as the time between viral acquisition by a mosquito on a viremic host and the transmission of viral infection to a susceptible host [43], is a key parameter in the control of WNV infection. Thus, mosquito immunity can act directly on this extrinsic incubation period, by limiting viral pathogenesis and dissemination to levels that are not detrimental for them. Indeed, recent studies from Carla Saleh’s lab have unraveled a very interesting mechanism taking place in mosquitoes to allow them to control viral pathogenesis. The viral genome is efficiently detected by Dicer-2 and then degraded by RNAi in mosquito cells. In addition, endogenous reverse transcriptases transcribe the viral genome or elements of the viral RNA into viral DNA, which is then integrated into the host genome or maintained as extrachromosomal DNA (episome) [44]. This mechanism relies on active endogenous retrotransposons in insect cells, which harbor important basal retrotranscriptase activity. The viral DNA is then transcribed into RNA and recognized by Dicer-2, thus re-engaging and amplifying the RNAi response [45,46]. This original mechanism controls the viral pathogenesis, allowing cell survival. The balance between antiviral immunity and viral escape that takes place in mosquitoes contributes to efficient viral transmission to a new host. Thus, mosquito cells carry a high viral load, notably into salivary glands [37,43], without displaying any symptoms. 

### 2.2. Antiviral Factors and Viral Countermeasures

In the context of WNV mosquito infection, only a few antiviral factors have been identified so far. In 2014, Yasunaga et al. performed a genome-wide RNAi screen in *D. melanogaster* that led to the identification of several WNV restriction factors [47]. They demonstrated that dRUVBL1 (pontin/Tip49, an ATPase), dRUVBL2 (reptin/Tip48, another ATPase), Tip60 (histone acetylase), and dXOP1 (embargoed, a nuclear export receptor) had antiviral activity against WNV in vivo in flies, and in vitro in mosquito cells (*Aedes aegypti* Aag2 cell line). dRUVBL1, dRUVBL2, and Tip60, which are all members of the Tip60 chromatin-remodeling complex, may play a role in the WNV antiviral transcriptional response by remodeling chromatin [48]. dXOP1 also participates in WNV restriction by mediating the nuclear export to the cytoplasm of the mRNA coding for the antiviral protein dALDOA (aldolase, glycolytic enzyme). Finally, the authors showed that dRUVBL1 and dXOP1, which are conserved from insects to mammals, restrict WNV infection in human cells (U2OS, HEK293T) and in mouse primary neurons [47].

Several strategies developed by viruses to overcome the mosquito antiviral response have been described in the literature [49,50,51]. For example, the WNV 3′-UTR encodes an miRNA-like small RNA that facilitates viral replication in mosquito cells, through the upregulation of GATA4 mRNA [52]. GATA4 belongs to a family of transcription factors notably involved in immune signalization. Furthermore, it was demonstrated in 2015 that the WNV 3′-UTR also encodes a subgenomic flavivirus RNA (sfRNA) that suppresses the RNAi response in *Culex* mosquitoes and enables WNV to infect the midgut [53]. As our understanding of mosquito immunity is growing, we can expect additional antiviral strategies that allow viral persistence and transmission to be uncovered.

## 3. Vertebrates: A General Insight into Anti-WNV Immunity

### 3.1. Immune Response to WNV Infection

In the skin, immune cells (in particular, skin-resident DCs and macrophages) are among the first cells encountered by viruses and they release proinflammatory cytokines and chemokines [54,55,56], which are important for the promotion and regulation of innate cell-mediated responses to WNV infection by natural killer cells (NKs) [57,58], polynuclear neutrophils [59], and γδ T-lymphocytes [60]. Furthermore, activated DCs and macrophages prime B- and T-lymphocytes, which are the main effectors of the adaptive immune response, coordinating the humoral and the cell-mediated immunity, respectively [61,62]. B-cells are responsible for the secretion of specific antibodies (Abs) that can neutralize or opsonize WNV by binding to viral epitopes. Moreover, some days after the onset of viral infection symptoms, B-lymphocytes produce immunoglobulins M (IgM). These Abs have a short-lasting activity in contrast with IgG, whose production occurs later than IgM but confers a long-lasting protection against a potential re-infection, as observed in in vivo studies [63,64,65,66]. T-lymphocytes also have a key role in the efficient adaptive response to WNV infection in humans. There are three major T-cell subsets, CD4+ T-cells (also known as helper T-lymphocytes), CD8+ T-cells, and regulator T-cells (Tregs). CD8+ T cells have been shown to be essential for the protection and clearance of WNV infection in mice [67,68,69]. In vivo, CD4+ T-cells coordinate the adaptive response by priming a specific B-cell response to WNV and sustain CD8+ cells activity [68,70,71], whereas Tregs modulate the immune response in order to keep the balance between an effective viral clearance and an exacerbated immune response to WNV infection that could be detrimental for the host [72]. 

The complement system has also been described to be part of the response against WNV infection [61]. This innate immune enzymatic cascade interacts with and senses pathogens, resulting in the formation of the membrane attack complex that directly lyses the pathogen or the infected cell. Besides its role in innate immunity, the complement enhances the humoral response and modulates T cell function [73]. Studies have highlighted the requirement of the complement activation pathways in the protection of mice against WNV infection, partly through their ability to induce a protective antibody response [74,75].

### 3.2. Innate Immunity

Innate immunity is the first line of defenses against viral infection, including WNV. All vertebrates have developed sophisticated strategies in order to detect intracellular pathogens, through the action of specialized receptors called pattern-recognition receptors (PRRs), which recognize specific molecular features called pathogen-associated molecular patterns (PAMPs). Among the many and diverse PRRs, which include C-type lectin receptors (CLRs) and NOD-like receptors (NLRs), this review will cover mainly two classes of PRRs: The toll-like receptors (TLRs), which are present on the plasma or the endosomal membranes, and the retinoic acid-inducible gene I (RIG-I)-like receptors (RLRs), which are cytoplasmic. The RLRs RIG-I and MDA-5 play a prominent antiviral function since they can sense viral RNAs and are ubiquitously expressed [61]. The sensing of PAMPs by PRRs leads to the activation of innate immune signaling cascades and to the nuclear translocation of transcription factors, in particular the nuclear factor-κB (NF-κB) and the interferon regulatory factors 3 and 7 (IRF3/7). Once translocated into the nucleus, NF-κB and IRF3/7 induce the expression of proinflammatory cytokines and type I IFN, respectively. Following transcription and translation, IFN-α/β are secreted and act in an autocrine and paracrine manner by interacting with interferon alpha/beta receptors (IFNARs). A second signaling cascade, called the JAK-STAT pathway, then induces the expression of hundreds of interferon-stimulated genes (ISGs) in order to establish an antiviral state. Another level of complexity to this system is that the level of ISG induction is dependent on many criteria, including the cell type, the type of IFN, its concentration, and stimulation kinetics. Furthermore, some ISGs are themselves components of the innate immune signaling pathways (RIG-I, MDA-5, IRF1, IRF7), while others are transcribed only through the activation of the JAK/STAT pathway (interferon-induced transmembrane proteins, IFITMs; IFN-induced proteins with tetratricopeptide repeats, IFITs) [76]. Finally, the most highly induced ISGs are not necessarily the most effective against a given virus, because the antiviral activity is not correlated with the magnitude of IFN induction. The establishment of the IFN-induced antiviral state consists in a complete cell reprogramming mechanism, in which some ISGs display a direct antiviral activity while others shape a hostile cellular environment, such as a general translation shutdown [77]. IFN-induced antiviral effectors can also be considered as restriction factors and can act at any step of the viral replication cycle. However, bona fide restriction factors are constitutively expressed, and their expression is either poorly or not further induced by IFN. These factors constitute the so-called intrinsic immunity [78]. 

WNV was shown to be sensitive to type I [79,80], type II [68], and type III IFN [81]. In addition, many papers have highlighted the requirement for components of innate immune signaling pathways for the control of WNV infection and the host antiviral response. Notably, among these components, RIG-I and MDA-5 [82,83,84,85], MAVS [86,87], Myd88 [88], and STING [89] were important for the immune control of WNV infection in human cells and in vivo in mice. Furthermore, many IRFs, such as IRF3 [90,91], IRF7 [91,92], and IRF5 [91,93], were demonstrated as being crucial for WNV restriction by mediating the IFN-dependent and/or IFN-independent response. Most restriction factors active against WNV identified so far are ISGs [94], thus confirming the importance of the IFN system upon WNV infection.

## 4. Birds: Immune Defenses against WNV Infection

### 4.1. Importance of Birds in the WNV Enzootic Cycle

Birds are the main reservoirs as well as the amplifier hosts of WNV. After the blood meal of an infected mosquito, WNV is able to replicate at high titers in birds, in particular in Passeriformes (house sparrows), Falconiformes (hawks), and Strigiformes (owls) [95,96]. Bird migration plays a key role in the global widespread of WNV infection and allows transmission beyond the living range of mosquitoes [97]. Usually, birds do not show any signs of infection, except for some bird species, such as members of the Corvidae family (crows, jays, and magpies), which can develop neurological symptoms and even die. WNV can even be responsible for massive bird outbreaks associated with fatal cases [98]. During the 2019 WNV transmission season, the Animal Disease Notification System (ADNS) of the European Commission reported 54 bird outbreaks in Europe [99]. As for other arboviruses, WNV can be detected in peripheral organs and tissues, including the brain, spleen, liver, lung, kidney, and heart, from naturally infected avian hosts [100,101,102].

### 4.2. Immune Response to WNV Infection

Upon WNV infection, house sparrows, hawks, and eagles can develop neutralizing antibodies at constant titers and neutralizing activity for 36 months, providing a long-lasting protection over multiple viral transmission seasons [103,104]. Maternally inherited WNV-specific antibodies (passive immunity) have also been detected in chickens [105], flamingo chicks [106], rock pigeons [107], and owls [108]. In addition to adaptive immunity, some cellular defenses against WNV infection have also been described in birds. The 2′-5′-oligoadenylate synthase pathway, for instance (OAS, see Section 5.2.3. OAS/RNase L) has been shown to participate in the control of WNV infection, notably in chickens [109,110]. Tag-El-Din-Hassan H. T. et al. showed that chicken OAS inhibits WNV replication [111], which may at least partly explain why chickens are more resistant to WNV infection than other bird species [112]. Given the general susceptibility of birds to WNV infection, they have been chosen as sentinel hosts in surveillance programs [113,114] in order to rapidly detect ongoing WNV outbreaks.

## 5. Immune Defenses against WNV Infection in Mammals

### 5.1. WNV Infection 

Flaviviruses induce systemic infections and can replicate in various tissues, including the brain, spleen, lymph nodes, kidney, and skin [115,116]. WNV can be transmitted to humans by organ transplantation [115,117], blood transfusion [118], or mosquito bites. Regarding the latter, skin represents the first replication site for the virus, where keratinocytes [119,120] and skin resident DCs [54,121] support active WNV replication. However, WNV is primarily described as a neurotropic virus that can induce neurological symptoms in humans, ranging from headache to encephalitis [122,123,124]. Indeed, WNV has the ability to infect neurons, the spinal cord, and the brain stem [22,23]. Two receptors belonging to the family of cell surface phosphatidylserine receptors present on microglia [125,126], TIM-1 (T cell/transmembrane, immunoglobulin, and mucin 1) and TIM-4, have been shown to significantly enhance WNV infection and may have an important role in WNV neurotropism [127]. The neuroinvasive potential of WNV has also been linked to N-linked glycosylation of its envelope protein [128] and seems to participate in the differential virulence between WNV lineage 1 and 2 [54,129,130,131].

### 5.2. Restriction Factors Targeting WNV 

#### 5.2.1. Protein Kinase R (PKR)

One of the first reported WNV restriction factor was PKR, also known as EIF2AK2. This dsRNA-dependent kinase, identified in 1990 [132], is activated by autophosphorylation when it binds dsRNA [133], such as WNV replication intermediates. In turn, PKR phosphorylates the translation initiation factor EIF2a, thus inhibiting viral and cellular RNA translation. Since WNV is a positive (+) ssRNA virus, its genome is rapidly translated from its genomic RNA to produce new virions, contrary to negative (-) ssRNA viruses for which genomes need to be first transcribed into (+) RNA before viral translation. Thus, PKR-mediated translational shutoff is an effective antiviral mechanism to restrict viral infection. In addition to its direct antiviral activity, PKR also acts as a dsRNA PRR and participates in the IFN response in infected cells [134]. Samuel M. A. et al. showed that PKR was able to limit mice mortality following WNV infection by restricting WNV replication in peripheral lymphoid tissues, and to inhibit viral replication in murine primary cortical neurons [135]. PKR-mediated restriction of WNV infection was confirmed a few years later in a model of HEK293T cells inducibly expressing individual ISGs [136].

#### 5.2.2. Viperin

Viperin (RSAD2) is a protein that inhibits a wide range of DNA and RNA viruses, like HCV [137], IAV (influenza A virus) [138], and WNV [136]. Via its amphipathic α helix, viperin localizes on the cytosolic face of the endoplasmic reticulum (ER) and inhibits protein secretion [139]. A study also showed that viperin associates with lipid droplets, which are intracellular lipid storage organelles important for the replication of many viruses, including HCV [140]. In plasmacytoid dendritic cells, viperin was found to localize within lipid bodies and to promote IRF7 nuclear translocation following TLR9 stimulation [141]. Jiang D. et al first identified viperin as an anti-WNV factor by screening the effect of a set of ISGs on viral replication [136]. In 2011, in vivo experiments strengthened the importance of viperin in WNV restriction [142]. Indeed, viperin^-/-^ mice are more susceptible to lethal WNV infection and neuroinvasion than wild-type mice (WT). There is more WNV replication in the cortex, spinal cord, and white matter, as well as a higher viral load in the brain of viperin^-/-^ mice than in control mice. Another in vivo study confirmed the antiviral activity of viperin against WNV and that viperin is required for WNV restriction in the spleen, the kidney, and the brain [142]. Interestingly, Lindqvist R. et al. demonstrated that viperin confers a region-specific protection against different neurotropic flaviviruses, including WNV, in the brain of infected mice [143]. In 2018, Gizzi A. S. et al. established that viperin can catalyze the formation of 3′-deoxy-3′,4′-didehydro-CTP (ddhCTP) nucleotides, which act as chain terminators for flaviviruses RNA-dependent RNA polymerases [144]. Since viperin is located at the cytoplasmic face of the ER, which is the replicative organelle for flaviviruses, it is thought to interfere with WNV replication by inhibiting RNA transcription and possibly also the assembly and budding of immature virions.

#### 5.2.3. OAS/RNase L

Although WNV induces high mortality rates in most laboratory inbred mice after peripheral inoculation, it was shown, surprisingly, that captured wild mice are resistant to WNV infection [145,146]. A single locus was shown to confer the phenotype of resistance/susceptibility encoding 2′-5′-oligoadenylate synthetases (2′-5′-OAS) [146]. Susceptible mice have a point mutation, introducing a pre-mature stop codon in exon 4 that generates a truncated form of the OAS1 protein isoform b (OAS1b-tr), whereas resistant mice have a normal copy of this gene (OAS1b-FL = OAS1b-full length) [147]. The Oas gene encodes an oligoadenylate synthetase that catalyzes the formation of 2′-5′ linked oligoadenylates [148] upon binding to dsRNA, such as flavivirus replication intermediates. These 2′-5′ oligos activate in turn the endoribonuclease L (RNase L), which degrades viral RNA into small RNAs. These are then recognized by RIG-I and MDA-5, thus activating innate immune signaling cascades and IFN-β production [149]. OAS1b and RNase L induce the degradation of viral genomes, the inhibition of protein secretion, and the activation of an immune response [150,151]. 

In 2007, a Cre-lox knock-in of the Oas1b resistance allele in a susceptible mouse model was shown to restore resistance to flavivirus infection, confirming that this gene is sufficient to confer WNV resistance [152]. Some years later, Lim J. K. et al. identified a single nucleotide polymorphism (SNP) in the Oas1 gene (rs10774671 SNP) that acts as a genetic risk factor for WNV infection in humans [153]. However, Bigham A. W. et al. were not able to find a significant requirement for the rs10774671 SNP in WNV infection but identified another SNP in the Oas1 gene, rs34137742, which was associated with an increased risk of encephalitis and paralysis upon human WNV infections [154]. More insight into the antiviral activity of OAS1b was provided in 2014, when Deo S. et al. demonstrated that two stem loops, SLII and SLIII, present in the WNV 5′-UTR, activate OAS1 in vitro [155]. Mutations in the dsRNA binding site of OAS1 confirmed the specificity of the interaction with WNV SLII/III. This result is in agreement with the fact that 5′- and 3′-UTR are highly structured non-coding regions that have important regulatory functions in viral replication [156]. One year later, the same group showed that WNV 3′-UTR was also able to specifically interact with and to activate OAS1 protein by a stem loop present in the 3′-UTR. The circularization of the WNV genome between 5′- and 3′-UTR by base pairing, which occurs during viral replication, was not found to be sufficient to protect WNV from OAS1 recognition in vitro. 

Concerning the involvement of RNase L in WNV resistance, a 2019 study from Madden J. C. et al. showed that RNase L was not critical [147]. Indeed, resistance phenotypes in RNase L^+/+^ and RNase L^-/-^ mice were similar, and RNase L activity in susceptible mice was not sufficient to inhibit infection or to increase the mouse survival rate. These results indicate that RNase L is not a required component for the OAS1b antiviral activity. The requirement of ABCF3, an ATP binding cassette protein part of a complex with OAS1b, for the anti-flavivirus activity mediated by OAS1b strengthens the results from Madden J. C. et al. [157]. It is therefore possible that ABCF3, rather than RNase L, acts in coordination with OAS1b to promote resistance to WNV infection.

#### 5.2.4. IFITMs

Interferon-induced transmembrane proteins (IFITMs) were among the first studied ISGs [158], and were particularly well characterized as in vitro IAV restriction factors [159]. IFITMs are type I and II IFN-induced membrane-associated proteins that localize at the plasma membrane as well as the membranes of endosomes and lysosomes. They can also be incorporated into viral particles, as shown for human immunodeficiency virus-1 (HIV-1) [160]. To date, five IFITM genes have been identified in humans, but only IFITM1, 2, and 3 are induced by IFN and involved in antiviral defenses [161]. The localization of IFITMs has been shown to be a key parameter for their activity. For example, mutations within the 20YXXΦ23 motif (endocytic signal) [162] that targets IFITM3 to endosomes led to its accumulation at the plasma membrane and restored infection by viruses that enter cells by endocytosis, such as IAV or human coronaviruses NL63 and 229E [162,163,164,165]. On the contrary, mutations in the endocytic motif enhanced the restriction of viruses directly entering cells at the plasma membrane, such as HPIV-3 and HIV-1 [166,167,168], highlighting the importance of IFITM3 localization for its broad-spectrum antiviral activity. Although IFITM1 and IFITM2 both display some antiviral activity [136], IFITM3 was found to be particularly essential for cellular defense and has therefore been more deeply characterized. 

In 2009, Brass A. L. et al. demonstrated that WNV is restricted by IFITMs and that IFITM3 overexpression drastically inhibited infection in A549 and U2OS cell lines, whereas IFITM depletion restored WNV infection [159]. More recently, Ifitm3^-/-^ mice were shown to be more susceptible to lethal WNV infection, thus demonstrating the role of IFITM3 as a viral restriction factor in an in vivo model of WNV infection [169]. 

IFITM-mediated restriction occurs at the step of viral entry into host cells, by inhibiting viral fusion at the plasma membrane or in endocytic vesicles. Emerging evidence suggests that IFITMs can block viral entry when expressed either in viral or cell membranes [160,163,170,171,172]. Whether from the virus or the cell side, the ability of IFITM proteins to alter lipid fluidity by disrupting intracellular cholesterol homeostasis is known to participate in the blockade of the fusion event occurring during viral entry [173]. Moreover, a 2019 study from Spence J. S. et al. showed that endocytic-localized IFITM3 fuses with incoming viral particles and enhances cargo trafficking to lysosomes, leading to lysosomal-mediated degradation of incoming viruses [174]. The involvement of IFITM3 palmitoylation, previously described as being important for its restriction activity [175], was also confirmed by this recent study. Altogether, these in vitro and in vivo data strengthen the importance of IFITM3 in restricting WNV, although a number of questions remain to be addressed, such as its possible impact on the immune adaptive response, as highlighted by Gorman M. J. et al. [169].

#### 5.2.5. ISG15/20

ISG15 and ISG20 derive their names from their molecular weight, respectively 15 kDa and 20 kDa, and their expression following IFN stimulation. ISG15, which was purified and characterized in 1984 [176], acts through an ubiquitin-like mechanism called ISGylation, which involves specific enzymes that catalyze the covalent conjugation of ISG15 to target proteins [177,178]. ISG15 has been reported to promote the IFN response by inhibiting PIN1-induced IRF3 ubiquitination [179] and to restrict infection by the arbovirus Sindbis virus [180]. Dai J. et al. showed in 2011 that ISG15 transcription is highly induced in WNV-infected cells compared to non-infected cells, using a model of mouse macrophage-like cells (RAW264.7 cell line) [181]. In addition, depletion of Isg15 in WNV-infected cells resulted in significantly higher viral loads as well as the upregulation of IFN-β transcription, indicating that ISG15 contributes to IFN-induced WNV restriction. 

ISG20 also displays antiviral activity against WNV, as Jiang D. et al. observed a significant decrease of infection by replicon-containing WNV-like particles (VLPs) in a model of HEK293T cells inducibly expressing individual ISGs [136]. However, the mechanism differs somewhat from ISG15 since ISG20 is a 3′ to 5′ exonuclease and has been shown to inhibit viral replication through viral RNA degradation [182,183]. More recent studies revealed other possible activities of ISG20 that do not implicate its exonuclease activity. For instance, Weiss C. M. et al. reported that its antiviral activity was at least partly due to the positive regulation of the IFN response, thus increasing the expression of other antiviral ISGs [184]. More recently, using VSV as a model RNA virus, Wu N. et al. proposed that ISG20 acts by decreasing protein translation from exogenous RNA in the absence of RNA degradation [185].

#### 5.2.6. IFITs

The mRNAs from higher eukaryotes, and some viral RNAs, have a ribose 2′-O-methylation on their 5′ guanosine cap [186]. This methylation, which is essential for RNA translation and RNA stability, defines two 5′-cap structures: The cap 0 structure without 2′-O methylation (usually found on viral RNAs) and the cap 1 structure with 2′-O-methylation (present on cellular mRNAs).

The 2′-O-methylated cap is recognized as the self by the host immune system. As a consequence, many viruses have either acquired such methylation activity or hijack cellular methyltransferases to modify their own mRNAs in order to evade innate immune recognition. This is particularly true for some viral genera that replicate in the cytoplasm, such as Flavivirus, and in the case of WNV, this activity is attributed to its NS5 methyltransferase activity (see Section 5.3.). Following viral infection or IFN treatment, there is a rapid induction of IFN-induced proteins with tetratricopeptide repeats (IFITs), which are classical ISGs [90,187]. In humans, this ISG family comprises IFIT1 (ISG56, P56), IFIT2 (ISG54, P54), IFIT3 (ISG60, P60, GARG-49), and IFIT5 (ISG58, P58), which all localize in the cytoplasm [188] and are known to homo- or heterodimerize. They share the ability to bind RNA and to directly interact with the translation initiation machinery, thus inhibiting viral RNA translation. In vivo, IFIT1/2/3 transcripts are strongly upregulated by WNV infection in the cerebellum of infected mice [189].

IFIT1 has been particularly well characterized and displays a potent antiviral activity towards several viruses, including WNV [190,191]. IFIT1 restriction is mediated through two mechanisms of action. 

First, the N-terminus and middle regions of IFIT1 have the ability to bind and recognize uncapped 5′-PPP RNA (5′-triphosphorylated RNA), a feature harbored by many viral genomes [192,193]. Indeed, uncapped 5′-PPP RNA can be generated during viral replication and sensed by PRRs, such as RIG-I or MDA-5. Uncapped 5′-PPP viral RNA recognition by IFIT1 leads to the assembly of an IFIT1/2/3 complex that either sequesters RNA away from an actively replicating RNA pool or promotes RNA degradation, thus inhibiting viral infection [192]. While WNV 5′-PPP RNA is usually hidden by its 5′-cap 0 structure, uncapping occurs during negative strand RNA synthesis, thus allowing recognition by innate immune sensors or by IFITs. Second, the IFIT1 C-terminus can inhibit translational initiation by binding to eukaryotic initiation factor 3 (eIF3) [194,195,196] and competing with eIF4E for binding to the 5′-end 2′-O-unmethylated RNA [196,197,198]. 

Szretter K. J. et al. suggested that IFIT1 does not contribute to the restriction of WNV in vivo, since the deletion of Ifit1 did not significantly enhance WNV pathogenesis in infected mice. However, IFIT1 did restrict a WNV mutant lacking 2′-O -methylation in MEFs, dendritic cells, and neurons from infected mice [199]. Similarly to IFIT1, IFIT2 has the ability to bind eIF3 [200] but also 5′-PPP RNA through its RNA binding domain [201]. The induced expression of murine IFIT2, whether upon IFN treatment or WNV infection, is dependent upon the phosphorylation of STAT1 on Ser-708 [202] catalyzed by IKKε. 

This post-translational modification was shown to be essential for WNV restriction in MEFs [203] and to contribute to the control of WNV infection in vivo, since IKKε^-/-^ mice display a more neurovirulent infection compared to WT-infected mice. In addition, Ifit2^-/-^ mice were more susceptible to lethal WNV infection than wild-type mice (92% vs. 38%) since IFIT2 restricts viral replication in the central nervous system (CNS; cerebellum, olfactory bulb). Thus, IFITs are important and complex restriction factors that in addition seem to display other activities than their antiviral one, like induction of apoptosis [204,205].

#### 5.2.7. IFI6, SC4MOL, DDX24, IFI44L, IFRD1, IL13RA1, MAFK, PAK3, and SAMD9L 

In a key study, Li J. et al. identified several WNV restriction factors by performing an shRNA (short hairpin RNA) screen in HeLa cells [206]. They showed that the depletion of DDX24 (an RNA helicase), IFRD1 (IFN-related development factor), IL13RA1 (α-subunit of the IL-13 receptor), MAFK (a transcription factor), PAK3 (a serine-threonine kinase), SAMD9L (sterile alpha motif domain containing protein 9 like), IFI44L (interferon-induced protein 44 like), IFI6 (interferon-induced protein 6), and SC4MOL (an endoplasmic reticulum protein) significantly increased WNV infection [206]. However, among all these antiviral effectors, only SC4MOL and IFI6 were shown to be sufficient to inhibit WNV replication. Although most of these proteins were previously not known to display any antiviral functions, IFI6 was characterized as one of the most important IFN-inducible antiviral effectors against flaviviruses [207,208]. The localization of IFI6 to the ER contributes to its antiviral activity by interfering with replication organelles’ membranes, thus perturbing viral replication, as it takes place in single-membrane invaginations localized on the ER and containing all the viral replication machinery [209].

#### 5.2.8. IFI27l2a

Another IFN-inducible antiviral effector against WNV has been identified in vivo in mice in 2016: IFI27l2a. There are three murine paralogs, with Ifi27l2a exhibiting the greatest induction following IFN treatment [152]. Ifi27l2a is constitutively expressed in peripheral organs, such as the lymph nodes, kidney, heart, and lung. Upon WNV infection, its expression is strongly upregulated notably in the brain, the spinal cord, the spleen, and the lymph nodes of infected mice [210]. Ifi27l2a deficiency increases the susceptibility to WNV infection in vivo and viral replication in the cerebellum and brain stem of Ifi27l2a^-/-^ mice, suggesting a protective role of IFI27l2a against WNV in the CNS, probably by limiting neuronal cell death [153]. Of note, IFI27l2a (ISG12b) belongs to the Ifi27 gene family that comprises four members in humans, of which only Ifi27 and Ifi6-16 are IFN inducible. 

#### 5.2.9. TRIM6/VAMP8

More recently, TRIM6, a tripartite motif (TRIM) protein with E3 ubiquitin ligase activity, was demonstrated to be involved in the control of the type-I IFN response and WNV replication in infected cells [211]. Specifically, TRIM6 was shown to facilitate the activation of the IKKε kinase, upstream of the nuclear translocation of IRF3, thus promoting the expression of ISGs, such as the Vesicle-associated membrane protein 8 (VAMP8). VAMP8 does not interfere directly with viral replication but rather potentiates IFN signaling by favoring JAK1 activation (JAK/STAT pathway). The identification of these new positive regulators of the IFN-mediated anti-WNV response could suggest that other host factors, which remain to be identified, also regulate this innate immune response.

Other TRIM proteins, such as TRIM5α and TRIM79α, have recently been shown to efficiently restrict flavivirus replication, but intriguingly, only some tick-borne and no mosquito-borne (such as WNV) flaviviruses are sensitive to their activity [212,213]. 

#### 5.2.10. SLFN11

The antiviral factor Schlafen 11 (SLFN11) was initially described as a HIV-1 restriction factor through its ability to bind transfer RNA (tRNA) and counteract their viral-induced upregulation, thus leading to a codon-biased translation of viral transcripts [214]. Considering its mode of action, it could be expected that SLFN11 might also act on other viruses, such as (+) ssRNA viruses. Indeed, the genome of (+) ssRNA viruses needs to first be translated by cellular polymerases into a polyprotein in order to generate new virions, so their replication would be more sensitive to translation impairment than (-) ssRNA viruses, which have their own RNA polymerase and transcribe directly the incoming genome. A recent paper from Valdez F. et al. demonstrated that SLFN11 restricts WNV replication in a human glioblastoma cell line (A172) that is highly susceptible to WNV infection [215]. WNV infection downregulates a small subset of tRNAs and this has been shown to lead to a better folding of viral proteins after translation [215], as it is the case during HIV-1 infection but on a wider subset of tRNAs. In this context, SFLN11 prevented the downregulation of these tRNAs by WNV, thus impairing viral translation and virion infectivity. As is the case for HIV-1, WNV restriction by SFLN11 is mediated through its N-terminal region, by a conserved mechanism that has been well described for SFLN13 [216]. This illustrates the fact that previously characterized restriction factors can have a broader activity spectrum than initially anticipated.

#### 5.2.11. RIPK3

The receptor-interacting protein kinase 3 (RIPK3), along with RIPK1, is an activator of necroptosis, a programmed cell death leading to plasma membrane disruption [217], and participates in inflammatory signaling [218]. In 2017, Daniels B. P. et al. showed that Rikp3^-/-^ mice are highly susceptible to neuroinvasive WNV infection. RIPK3 was required for the neuroimmune control of WNV infection as it drives the expression of proinflammatory chemokines in neurons through its kinase activity and that of RPK1 [219]. Despite RIPK3 being involved in necroptosis, its restrictive effect on WNV replication is independent of necroptosis-induced cell death, since necroptosis was similar in primary neuronal cultures and from WT and Ripk3^-/-^ mice. In 2019, the same group demonstrated that RIPK3 was also involved in the restriction of neuronal ZIKV infection but through a different mechanism [220]. Eventually, a recent study highlighted the fact that the implication of RIPK3 in flaviviral infections might be more complex, since RIPK3 was shown to inhibit IFI44L expression and to increase JEV replication in neurons [221].

#### 5.2.12. Other Restriction Factors

A *Nature* paper from Schoggins J. W. et al. identified several ISGs active against different viruses, including WNV, based on a large-scale screen using GFP reporter viruses [207]. In this study, IRF1, HSPE, IFITM3, RIG-I, MDA-5, C60orf150, NAMPT, and PHF15 were described as WNV antiviral factors. However, a corrigendum was released a few years later indicating that the WNV-GFP stock used in this study was in fact a VEEV-GFP (Venezuelan equine encephalitis virus) stock [222]. Thus, in this work, all the identified ISGs supposedly acting against WNV were in fact targeting VEEV. Nevertheless, because some of these ISGs were found to be effective antiviral factors against other flavivirus, like YFV [207], it would be interesting to revisit the potential activity of these factors on WNV replication.

The main restriction factors interfering with WNV infection are depicted in red and a concise representation of the canonic innate immune signalization cascade involving RIG-I/MDA-5 and IRF3 is presented in blue. 

This innate immune pathway starts with the recognition of viral dsRNA by the cytoplasmic RNA sensors RIG-I and/or MDA-5. Once activated, RIG-I and MDA-5 associate with the mitochondrial antiviral signaling protein, MAVS [223], which activates the downstream components TBK1 and IKKε, leading to the phosphorylation and dimerization of IRF3. Phosphorylated IRF3 translocates in the nucleus and induces type I IFN gene transcription. In turn, autocrine and/or paracrine IFN signalization induces an antiviral state by activating the expression of ISGs, some of which act as potent restriction factors against WNV (in red). During infection, WNV particles attach on host cell and enter via clathrin-mediated endocytosis. Endosomal acidification leads to viral fusion with the endosomal membrane, a step that can be inhibited by IFTIMs [136,159,169,174]. During uncoating, viral RNA can then be detected by OAS1b [147,151,152,155,157], leading to the activation of RNase L, which degrades viral RNA into small RNAs. While ISG20 has also the ability to degrade viral RNA [182,183], viperin catalyzes the formation of ddhCTP nucleotides, which act as chain terminators for the viral RNA polymerase NS5, thus inhibiting viral transcription [144]. Viperin has also been reported to prevent viral replication and assembly in the endoplasmic reticulum [137,140,142]. However, viral protein synthesis seems to constitute a favored step for restriction, since the translation of viral RNA is inhibited by several antiviral effectors, such as PKR [135,137], IFITs [190,192,199,200,201], ISG20 [184,185], or SFLN11 [215]. Given that the precise antiviral mechanism of IFI27l2a, TRIM6/VAMP8, IFI6, DDX24, IFI44L, IFRD1, IL13RA1, MAFK, PAK3, SAMD9L, SC4MOL, and RIPK3 is still unclear, they are not represented in this diagram but are indicated in Table 1.

### 5.3. Viral Countermeasures

#### 5.3.1. Evasion from Innate Immune Recognition

WNV, like other flaviviruses, replicates within replication compartments. These organelles, localized at the ER, act as a physical barrier to hide dsRNA replication intermediates from detection by RLRs [31,224]. In addition to this physical evasion, WNV has also evolved an RNA modification strategy to escape from innate immune sensing by MDA-5 as well as from restriction by IFITs. Indeed, WNV NS5 possesses 2′-O-methylation activity that is able to modify the 5′-cap structure of viral mRNA [225]. Since 2′-O-methylation, present on cellular mRNAs, is recognized as the self by MDA-5, this activity contributes by subverting immune sensing [186]. Moreover, such viral 2′-O-methylation prevents the binding of IFIT1 to translation initiation factors, thus avoiding WNV restriction by IFIT1 in vivo [199,226,227].

#### 5.3.2. Direct Inhibition of Innate Immune Sensors

Besides the capacity of WNV to evade immune detection, almost all its viral proteins have evolved as direct antagonists of innate immune sensors. In particular, WNV NS1 plays an important role in immune evasion. WNV NS1 inhibits complement activation by binding the regulatory protein factor H (fH) [228]. In turn, fH binding induces the degradation of C3b and attenuates the formation of the C5b-9 membrane attack complex involved in the immune defense related to the complement system. Furthermore, recent studies have unraveled new evasion functions for NS1. First, in 2014, it was discovered that the α/β domain of NS1 shared similarities with the helicase domains of RIG-I and MDA-5 [229]. Such a similarity was proposed to mimic the RLRs structure in order to antagonize the innate immunity. In 2017, Zhang H.-L. et al. established that NS1 directly interacts with RIG-I and MDA-5, causing their proteasomal degradation [230]. In addition, NS1 was able to block IRF3 phosphorylation and nuclear translocation and to inhibit TLR3 signaling [231]. All these mechanisms could synergize WNV NS1 antagonism of IFN-β production. Recently, WNV NS3 has also been shown to inhibit RIG-I-induced signaling through a phosphomimetic 14-3-3ε-binding motif on NS3, the RxEP motif, thus blocking 14-3-3ε-induced RIG-I translocation to mitochondrial MAVS [232]. 

Concerning NS4, two studies of Liu W. J. et al. demonstrated that WNV NS4A suppressed IFN-β promoter activation, therefore impeding ISGs’ expression [130,233]. Moreover, NS4B inhibits TBK1 activation that is required for IRF3 phosphorylation and nuclear translocation [234]. WNV E protein is also part of viral evasion as it inhibits the RIP1 kinase, a positive RIG-I regulator, thus blocking the dsRNA-induced IFN response [235]. 

In addition to these viral protein-based evasion mechanisms, a 2008 study revealed that WNV encodes an sfRNA (subgenomic flaviviral RNA) derived from its 3′-UTR that contributes to type-I IFN response evasion in MEFs [236]. Furthermore, it was demonstrated that this WNV non-coding sfRNA is essential for viral-induced cytopathicity in Vero cells and pathogenicity in mice [237].

#### 5.3.3. Direct Inhibition of IFN Signaling

Direct inhibition of IFN signaling is another efficient viral evasion strategy used, for example, by Ebola virus [238], DENV [239], or ZIKV [240]. In this way, many studies have demonstrated that WNV NS4B is able to block the phosphorylation of both Janus kinase 1 (JAK1) and Tyrosin kinase 2 (TYK2), an essential step for STAT activation and establishment of the antiviral state [241,242,243]. In 2010, Mansflied K. L. et al. showed that this viral protein was also able to upregulate SOCS1 and 3 (suppressors of cytokine signaling 1 and 3) expression, thus dampening JAK1 activation and blocking IFNAR-dependent signaling [244]. Evans J. D. et al. also confirmed that WNV infection induces IFNAR degradation in HeLa and Vero cells [245]. 

Additionally, besides its methyltransferase activity, WNV NS5 can block STAT1/2 activation, thus abrogating IFN signaling [239,246], and by disrupting HSP90 chaperone activity on JAKs, NS5 was shown to broadly inhibit JAK/STAT signaling during WNV infection [247].

Finally, in 2012, Suthar M. S. et al. confirmed that structural and nonstructural WNV proteins take part in this general control of the IFN response pathway thanks to the generation and characterization of WNV chimeric viruses. The reported infectious WNV clones displayed comparable biological activity as well as in vivo pathogenicity in C57BL/6 mice when compared to parental WNV isolates [248].

## 6. Conclusions

**Figure 2:**
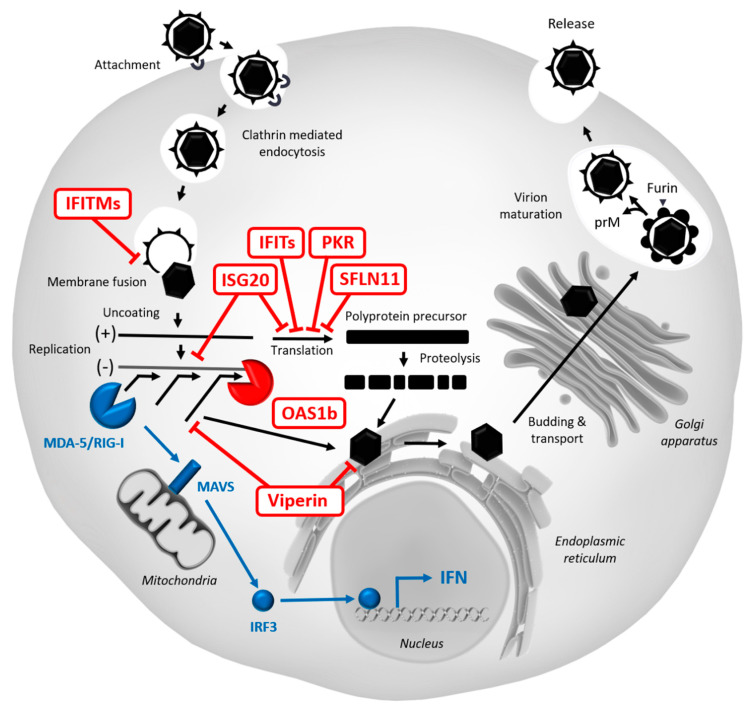
Restriction of WNV replication in human cells.

In this review, we highlighted the arms race that takes place between the control of infection and WNV evasion from the innate immune system in mosquito and human cells. Our understanding of the mechanisms involved in WNV restriction (Figure 2, Table 1) and viral evasion have considerably expanded over the past decade, thanks in particular to in depth in vivo studies and large-scale genetic screens. High-throughput screenings, in particular, like the one performed by Li J. et al. [206], led to the identification of previously unknown restriction factors. Nevertheless, further work is needed to confirm their activity in human primary cells and their in vivo relevance, and to unravel their mechanism of action. 

Furthermore, since WNV is responsible for increasingly frequent and important human outbreaks, we can reasonably believe that competent vectors will continue to spread around the world, thus additional fundamental studies are more than ever required. In particular, in vitro studies in more relevant human cells are lacking and will be important to better understand immune control, CNS protection, and viral evasion. Such knowledge will provide a basis to design therapeutic strategies in order to block viral replication, boost innate immune recognition/response, or detect resistant WNV variants with increased ability to counteract the immune system.

## Figures and Tables

**Table 1:** WNV restriction factors in mammalian cells: affected step of the viral replication cycle, mechanism, and models.

Restriction Factor	Affected Step	Mechanism	Model	References
PKR	Translation	Phosphorylation of the translation initiation factor EIF2a	Human cells & Mice	[135,137]
Viperin	Assembly Secretion	Catalyzes the formation of chain terminators for flaviviral RNA polymerases	Human cells & Mice	[137,139,142,144]
Oas1b	Sensing	Catalyzes the formation of 2′-5′-oligos / Belongs to an antiviral complex	Mice	[147,151,152,155,157]
IFITM2	Entry	Inhibits viral fusion with host membranes	Human cells	[136]
IFITM3	Entry	Inhibits viral fusion with host membranes	Human cells & Mice	[136,159,169,174]
ISG15	Replication	Up-regulation of the IFN response	Human & mice cells	[136,181,184]
ISG20	Replication, translation	Viral RNA degradation, translation inhibition, IFN response up-regulation	Human cells & mice	[182,183,184,185]
IFITs	Translation	Recognition of the 5′-end 2′-O-unmethylated RNA and 5′-PPP RNA / Binds eIF3 / Competes with eIF4E	Mice	[190,192,199,200,201]
IFI27l2a	Unknown	Unknown	Mice	[210]
TRIM6/VAMP8	Unknown	Regulation of the IFN response	Human cells	[211]
Schlaffen 11	Translation	Regulation of tRNA abundance	Human cells	[215]
IFI6	Unknown	Interferes with the membrane of replication organelles	Human cells	[206,207,208]
DDX24	Unknown	Unknown	Human cells	[206]
IFI44L	Unknown	Unknown	Human cells	[206]
IFRD1	Unknown	Unknown	Human cells	[206]
IL13RA1	Unknown	Unknown	Human cells	[206]
MAFK	Unknown	Unknown	Human cells	[206]
PAK3	Unknown	Unknown	Human cells	[206]
SAMD9L	Unknown	Unknown	Human cells	[206]
SC4MOL	Unknown	Unknown	Human cells	[206]
RIPK3	Unknown	Promotes inflammatory chemokines expression	Mice	[219]
